# Microbiota inoculum composition affects holobiont assembly and host growth in *Daphnia*

**DOI:** 10.1186/s40168-018-0444-1

**Published:** 2018-03-22

**Authors:** Martijn Callens, Hajime Watanabe, Yasuhiko Kato, Jun Miura, Ellen Decaestecker

**Affiliations:** 1 Aquatic Biology, Science and Technology, IRF Life Sciences, KU Leuven, Campus Kortrijk, E. Sabbelaan 53, 8500 Kortrijk, Belgium; 2 Centre d’Ecologie Fonctionelle Evolutive, CNRS Montpellier, UMR 5175, 1919 route de Mende, 34293 Montpellier CEDEX 5, France; 3 Bioenvironmental Science, Osaka University, Yamadaoka, Suita, Osaka, 565 0871 Japan

**Keywords:** *Daphnia*-microbiota interactions, Antibiotic exposure, Holobiont assembly, Microbiota transmission

## Abstract

**Background:**

Host-associated microbiota is often acquired by horizontal transmission of microbes present in the environment. It is hypothesized that differences in the environmental pool of colonizers can influence microbiota community assembly on the host and as such affect holobiont composition and host fitness. To investigate this hypothesis, the host-associated microbiota of the invertebrate eco(toxico)logical model *Daphnia* was experimentally disturbed using different concentrations of the antibiotic oxytetracycline. The community assembly and host-microbiota interactions when *Daphnia* were colonized by the disturbed microbiota were investigated by inoculating germ-free individuals with the microbiota.

**Results:**

Antibiotic-induced disturbance of the microbiota had a strong effect on the subsequent colonization of *Daphnia* by affecting ecological interactions between members of the microbiota. This resulted in differences in community assembly which, in turn, affected *Daphnia* growth.

**Conclusions:**

These results show that the composition of the pool of colonizing microbiota can be an important structuring factor of the microbiota assembly on *Daphnia*, affecting holobiont composition and host growth. These findings contribute to a better understanding of how the microbial environment can shape the holobiont composition and affect host-microbiota interactions.

## Background

Animal tissues in contact with the external environment, such as the surface of the body and the gut epithelium, are colonized by complex communities of microorganisms, collectively called the microbiota. At one extreme, gut symbionts can be directly transferred from mother to offspring, but most of the time they are randomly picked up from the environment [1]. In general, the gut microbiota is a multilayered structure, composed of both a core microbiota under host genetic and immune control and a flexible pool of microbes modulated by the environment [2, 3]. Although hosts can be colonized by opportunistic food-related or widespread environmental taxa, they are often directly or indirectly colonized by microbiota released in the environment by conspecifics [4]. In many species, the horizontal transmission between conspecifics is facilitated through different behaviors such as a gregarious lifestyle, coprophagy, trophallaxis, and parental care. This allows colonization of the host by an appropriate set of symbionts and results in the formation of the holobiont: the entity that comprises the host and all of its symbiotic microbes [5, 6].

It is essential that a host is colonized by an appropriate set of symbionts, as the microbiota provide beneficial services to the host. These services are provided through a variety of mechanisms such as enhanced food digestion [7] and uptake [8], the production of essential nutrients [9], detoxification of harmful substances [10], increased resistance to infection through colonization resistance [11], and enhanced host development and behavior through interactions with the host metabolism [12, 13]. Acquisition of an inadequate, inappropriate, or disrupted microbiome can, on the other hand, negatively impact the host’s fitness. This can occur either indirectly through competition with the beneficial microbiota or directly through, e.g., the production of bioactive metabolites that are detrimental to the host’s health [14, 15]. Therefore, community composition of the established microbiota can have a profound impact on the fitness of its host.

Many factors can, however, influence the community composition of the microbiota that becomes established on the host [3]. Processes governing the microbiota assembly seem to be strictly regulated in some species, strongly reducing inter-individual variation [16]. In other species, multiple factors contribute to inter-individual differences in the microbiota community composition [17]. The available environmental pool of microbes will be the first determinant of which symbionts can potentially colonize the host. These microbes will then be selectively recruited through interactions with the host and the already established microbiota [18]. Several studies have shown that the genetic background, developmental stage, and diet of the host can be important structuring factors of the microbiota [19, 20]. Furthermore, biotic interactions within the microbiota can determine the establishment of specific symbionts. Early colonizers can alter their direct environment, either allowing the settlement of other species through facilitation, exemplified by the occurrence of syntrophic interactions in microbiota communities [21, 22]. Although some studies have already addressed the effect of differences in the environmental pool of colonizers on microbiota assembly [16], results are sometimes contradicting and experimental data remains scarce. Furthermore, it is often not clear what the consequences are for the host’s fitness.

Here, we addressed this issue by inducing disturbance in the microbiota of *Daphnia magna* by exposure to the antibiotic oxytetracycline (OTC), a broad-spectrum protein synthesis inhibitor commonly used in aquaculture. Bacterial taxa are known to strongly differ in their susceptibility to oxyteracycline, so this antibiotic is expected to cause concentration-dependent changes in the microbiota community composition and function. These disturbed microbiota communities were subsequently used to colonize germ-free *D. magna*, and the microbiota assembly and host functioning were characterized. Antibiotics are well known for their capacity to induce disturbances that affect both the microbiota composition and internal ecological dynamics of the microbiota, which in turn can have an effect on host fitness [22, 23]. For example, a number of clinical disorders are found to be associated with disturbances in the gut microbiota due to antibiotic intake. *D. magna* is a widely used model organism in ecology, ecotoxicology, and evolution due to its short generation time, clonal reproduction, and ease of experimental manipulation [24]. Furthermore, several studies have already shown that the fitness of *D. magna* is highly dependent on its associated microbiota. In *D. magna*, growth, survival, and reproduction is strongly reduced in germ-free individuals [25–27]. Short-term exposure to the antibiotic trimethoprim was shown to negatively affect host growth by decreasing the digestion and incorporation of food [28], and the microbiome was estimated to play an important role in the detoxification of harmful algae [29]. Host-microbiota interactions within *D. magna* are known to be highly specific, e.g., only certain strains of the genus *Limnohabitans* are able to recover the fitness of germ-free individuals after re-inoculation [30].

In this study, three different questions are addressed. First, what is the impact of exposing the *Daphnia-*associated microbiota to different levels of antibiotic-induced disturbances? Here, a concentration-dependent shift in community composition is expected, with a reduction in susceptible taxa alongside an increase in resistant taxa upon increased antibiotic exposure. Second, how does a different degree of disturbance of the microbiota affect the subsequent colonization of germ-free individuals? Will the microbial community restore to its initial composition or does disturbance of the microbiota inoculum affects the colonization and community composition of the microbiota on *Daphnia*? Third, are host-microbiota interactions affected when hosts are colonized with microbiota exposed to different degrees of antibiotic-induced disturbance? Given the highly specific interactions between *Daphnia* and its microbiota, it seems plausible that an altered microbiota community composition can also affect host performance. To answer these questions, the temporal change in the *Daphnia* microbiota exposed to different oxytetracycline concentrations was investigated. Subsequently, germ-free *Daphnia* were inoculated with the microbiota extracted from these exposed populations and community assembly, and host growth were characterized.

## Results

### Effect of OTC exposure on *Daphnia*-associated microbiota

#### Bacterial load

**Fig. 1:**
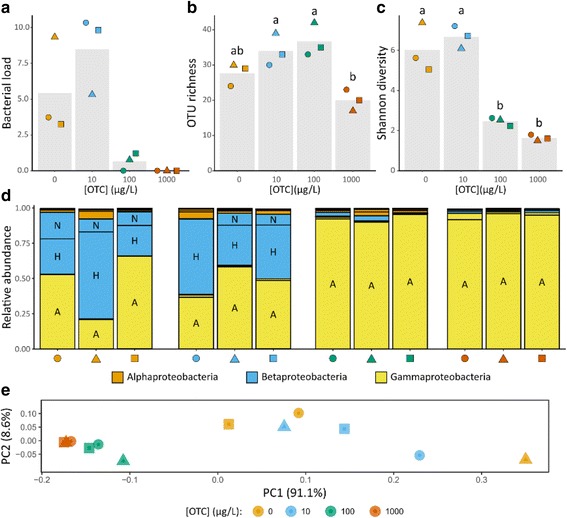
Effect of different concentrations of OTC on *Daphnia* microbiota after 7 days of exposure. In all graphs, individual populations are indicated with the same combination of color (OTC concentration) and shape (different populations within a specific OTC concentration). **A**–**C** Bacterial load, OTU richness, and Shannon diversity. Bars indicate mean values for each OTC concentration; points indicate specific values for each population. **B**, **C** Letters above bars indicate significant differences between OTC concentrations at 5% as determined by a Tukey HSD post hoc test. **D** Relative abundance of OTUs belonging to the Proteobacteria; color indicates proteobacterial class and letters inside the bars indicate specific OTUs (A = *Acinetobacter* sp., H = *Hydrogenophaga* sp., N = Neisseriaceae sp.). **E** PCoA of *Daphnia* microbiota in different populations using weighted Unifrac distances

**Fig. 2:**
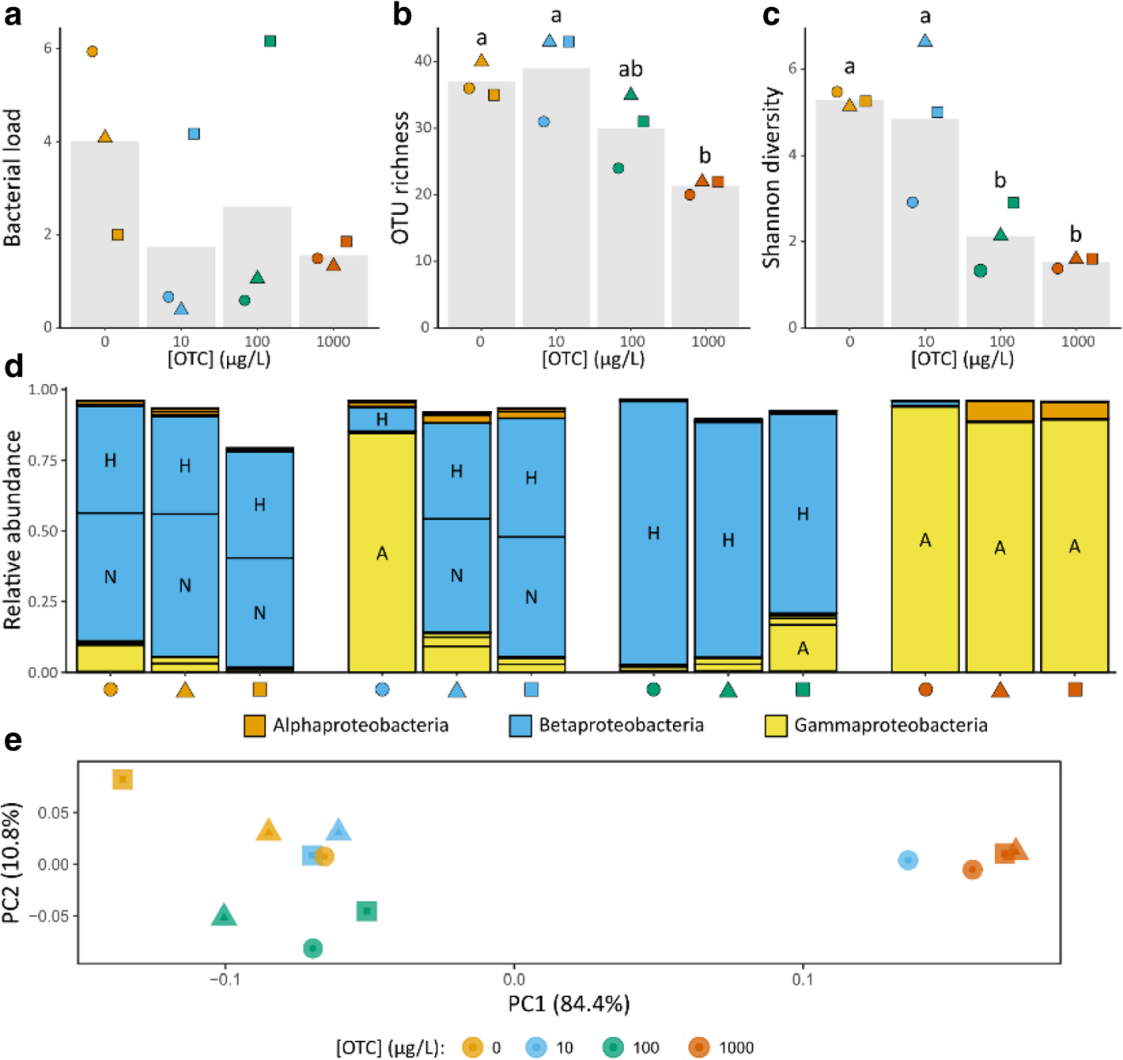
Effect of different concentrations of OTC on *Daphnia* microbiota after 23 days of exposure. In all graphs, individual populations are indicated with the same combination of color (OTC concentration) and shape (different populations within a specific OTC concentration). **A**–**C** Bacterial load, OTU richness, and Shannon diversity. Bars indicate mean values for each OTC concentration; points indicate specific values for one population. **B**, **C** Letters above bars indicate significant differences between OTC concentrations at 5% as determined by a Tukey HSD post hoc test. **D** Relative abundance of OTUs belonging to the Proteobacteria; color indicates proteobacterial class and letters inside the bars indicate specific OTUs (A = *Acinetobacter* sp., H = *Hydrogenophaga* sp., N = Neisseriaceae sp.). **E** PCoA of *Daphnia* microbiota in different populations using weighted Unifrac distances

An overall significant effect of OTC concentration on the bacterial load was found after 7 days of exposure (*X*^2^_3_ = 9.26, *p* < 0.05). At this point, the number of bacteria was below the detection threshold in all populations receiving 1 mg L^−1^ OTC (Fig. 1A). Also, the bacterial load in the populations receiving 100 μg OTC L^−1^ (0.66 ± 0.62) was considerably lower than the populations receiving no OTC (5.44 ± 1.96) and 10 μg OTC L^−1^ (8.49 ± 2.76). After 23 days of exposure, there was, however, no significant effect of OTC concentration on bacterial load (*X*^2^_3_ = 2.74, *p* = 0.43) (Fig. 2A). For populations receiving the same OTC concentration, a significant increase in bacterial load between 7 and 23 days of exposure in the populations receiving 1 mg OTC L^−1^ (*X*^2^_1_ = 3.86, *p* < 0.05) and a significant decrease in bacterial load in the control populations (*X*^2^_1_ = 3.86, *p* < 0.05) was observed.

#### Community composition

After 7 days of exposure, the *Daphnia* microbiota community in all three populations that were not exposed to OTC had an average of 27.6 ± 3.2 OTUs (Fig. 1B) and was mainly composed of *Acinetobacter* sp., *Hydrogenophaga* sp., and an OTU belonging to the family Neisseriaceae. There was, however, some variation in the relative abundance of these OTUs between different replicates, with either *Acinetobacter* sp. or *Hydrogenophaga* sp. being dominant (Fig. 1D). Populations exposed to 10 μg OTC L^−1^ had on average 34.0 ± 4.6 OTUs and a similar composition as the populations that were not exposed to OTC. However, Neisseriaceae sp. was present at a very low abundance (0.4%) in one of the replicates that was exposed to 10 μg OTC L^− 1^. At a concentration of 100 μg L^−1^, exposure to OTC had a very strong effect on the microbiota community composition, with *Acinetobacter* sp. being dominant at a relative abundance > 90% in all populations. The abundance of *Hydrogenophaga* sp. and Neisseriaceae sp. was furthermore strongly reduced. Although OTU richness remained high at 100 μg OTC L^−1^ (36.7 ± 4.7), there was a significant decrease in Shannon diversity compared to populations receiving no OTC or 10 μg OTC L^−1^ due to the strong dominance of *Acinetobacter* sp. (Tukey post-hoc p-adj. < 0.05) (Fig. 1C). Populations receiving 1 mg OTC L^−1^ showed a similar response to those receiving 100 μg OTC L^−1^ with *Acinetobacter* sp. being dominant. However, with an average of only 20.0 ± 3.0 OTUs, richness was significantly lower than populations receiving 100 μg OTC L^−1^ (Tukey post-hoc p-adj. < 0.05).

After 23 days, *Hydrogenophaga* sp. and Neisseriaceae sp. were codominant in all populations that were not exposed to OTC. Compared to the 7-day-old populations, there was a strong decrease in the relative abundance of *Acinetobacter* sp. (Fig. 2D) combined with an increase in the average number of OTUs (37.0 ± 2.6). In the populations exposed to 10 μg OTC L^−1^, two populations showed a similar response to those receiving no OTC, with an increase in the relative abundance of Neisseriaceae sp. and a decrease in *Acinetobacter* sp. One population, however, showed a strong increase of *Acinetobacter* sp. alongside a reduction in *Hydrogenophaga* sp. Microbiota communities in all populations receiving 100 μg OTC L^−1^ showed a drastic shift from being dominated by *Acinetobacter* sp. after 7 days of exposure to dominance by *Hydrogenophaga* sp. after 23 days of exposure. Neisseriaceae sp., on the other hand, was undetectable or present at a very low abundance in these populations. Shannon diversity remained significantly lower in populations exposed to 100 μg OTC L^−1^ than populations not exposed to OTC or to 10 μg OTC L^−1^ (Fig. 2C). Ordination furthermore shows that the microbiota community composition in populations exposed to 100 μg OTC L^−1^ for 23 days was more similar to those receiving no OTC or 10 μg OTC L^−1^ than to populations receiving 1 mg OTC L^−1^ (Fig. 2E). The microbiota communities in populations receiving 1 mg OTC L^−1^ remained relatively stable, with *Acinetobacter* sp. still being the dominant OTU.

### Assembly of microbiota communities after inoculation

**Fig. 3:**
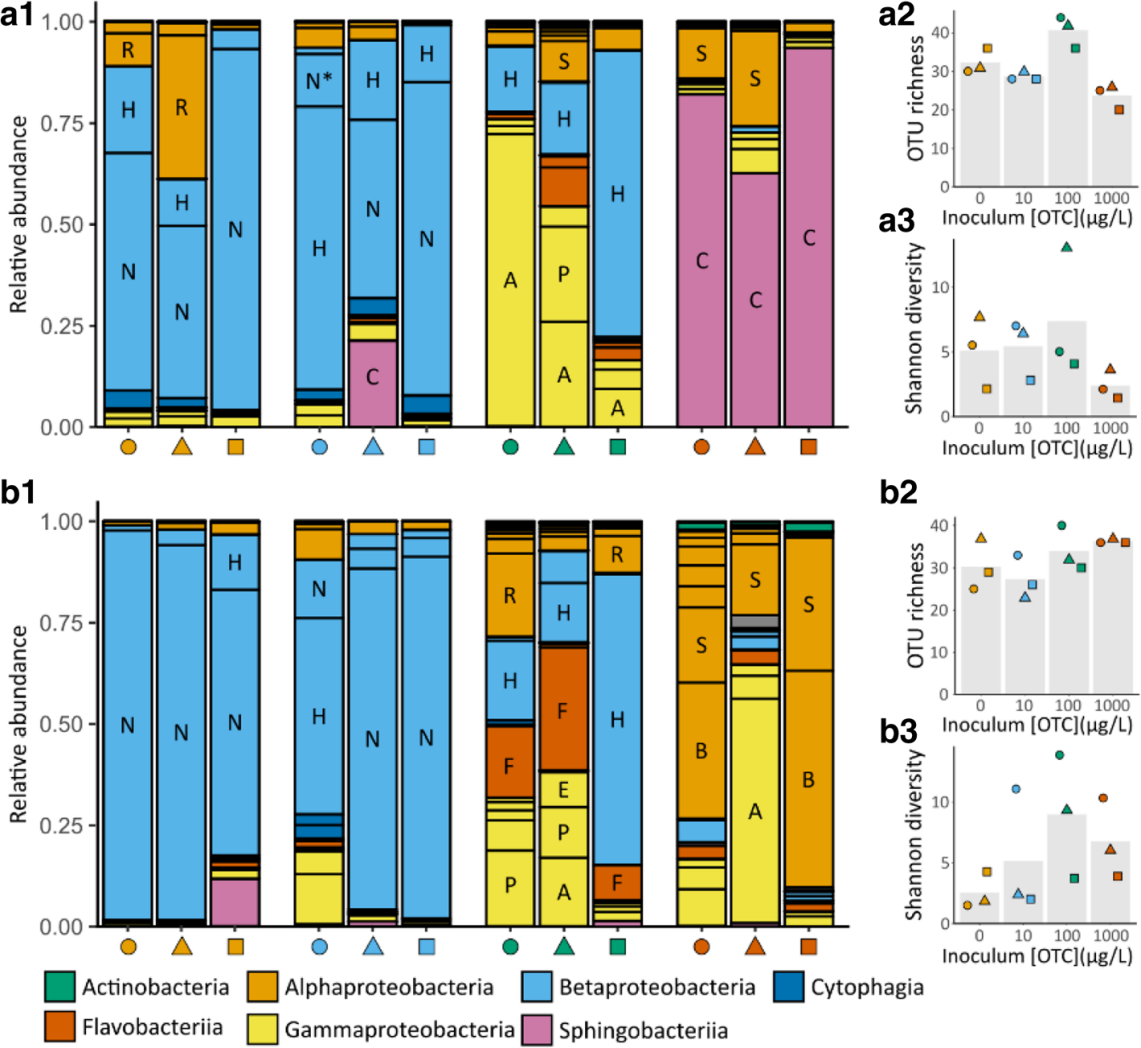
Assembly of the *Daphnia* microbiota after inoculation with microbiota exposed to different concentrations of OTC. The upper panel **A1**–**A3** shows the results for *Daphnia* inoculated with microbiota exposed for 7 days; the lower panel **B1**–**B3** shows the results for *Daphnia* inoculated with microbiota exposed for 23 days. In all graphs, individual populations are indicated with the same combination of color (OTC concentration) and shape (different populations within a specific OTC concentration). **A1**, **B1** Relative abundance of different OTUs in the *Daphnia* microbiota. Colors indicate the class to which the OTU belongs; major OTUs are indicated with a letter inside the bar (A = *Acinetobacter* sp., B = *Bosea* sp., C = Chitinophagaceae sp., E = *Escherichia/Shigella* sp., F = *Flavobacterium* sp., H = *Hydrogenophaga* sp., N = Neisseriaceae sp., P = *Pseudomonas* sp., R = Rhodobacteriaceae sp., S = *Shinella* sp.). **A2**, **A3**, **B2**, and **B2** OTU richness and Shannon diversity of *Daphnia* microbiota inoculated with microbiota that was exposed to different concentrations of OTC. Bars indicate mean values for each OTC concentration; points indicate specific values for one population

Germ-free *Daphnia* were inoculated with microbiota communities extracted from *Daphnia* exposed to different degrees of OTC-induced disturbance characterized in the previous section, and colonization and host growth of these inoculated *Daphnia* were investigated. The microbiota community composition on *Daphnia* after inoculation differed strongly from the composition of inoculum that was administered. Overall, the microbiota of *Daphnia* given an inoculum from populations that were not exposed to OTC or exposed to 10 μg OTC L^−1^ had a high relative abundance of Neisseriaceae sp., where it became the dominant OTU in the microbiota of several inoculated *Daphnia* (Fig. 3A1, B1). The relative abundance of Neisseriaceae sp. on *Daphnia* after inoculation seemed to depend, in part, on its abundance in the inoculum. Other prominent OTUs present in some of the microbiota on *Daphnia* inoculated from these same populations were *Hydrogenophaga* sp., Rhodobacteriaceae sp., and Chitinophagaceae sp. In *Daphnia* colonized from inocula exposed to 100 μg OTC L^−1^, Neisseriaceae sp. was either absent or present at a very low abundance (< 0.4%). Furthermore, while the microbiota community of some *Daphnia* was dominated by one OTU (either *Acinetobacter* sp. or *Hydrogenophaga* sp.; Shannon index ≤ 5), other microbiota communities had a higher evenness, with several OTUs present at an intermediate abundance (*Acinetobacter* sp., *Escherichia/Shigella* sp., *Flavobacterium* sp., *Hydrogenophaga* sp., *Pseudomonas* sp., Rhodobacteriacaeae sp., *Shinella* sp.; Shannon index > 9). When *Daphnia* were inoculated with microbiota that had been exposed to 1 mg OTC L^−1^, there was a strong difference in community composition depending on the length the inoculum was exposed to OTC. The microbiota of *Daphnia* for which the inoculum had been exposed for 7 days to 1 mg OTC L^−1^ was mainly dominated by Chitinophagaceae sp., with also higher abundances of *Shinella* sp. in some microbiota communities. In contrast, on *Daphnia* inoculated with microbiota exposed for 23 days to 1 mg OTC L^−1^, Chitinophagaceae was either absent or present at a very low abundance (< 0.1%). In these microbiota communities, the most abundant OTUs were *Acinetobacter* sp., *Bosea* sp., and *Shinella* sp. OTU richness in these *Daphnia* was also higher than in those inoculated with microbiota exposed for 7 days to OTC.

### Effect of inoculated microbiota on host growth

**Fig. 4:**
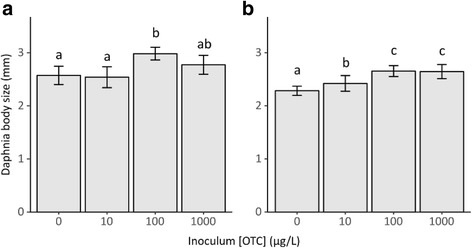
Body size of six-day-old *Daphnia* inoculated with microbiota from populations which were exposed for a different length (graph **a** 7 days; graph **b** 23 days) to different concentrations of OTC (no OTC, 10 μg L^−1^, 100 μg L^−1^ or 1 mg L^−1^). Letters above the bars indicate differences within an exposure time as determined by a Tukey HSD post hoc test. Different letters are significant at 5%

The OTC concentration to which the microbiota inoculum was exposed had a significant overall effect on *Daphnia* growth after both 7 days of exposure (*F*_3,8_ = 12.24; *p* < 0.05) and 23 days of exposure (*F*_3,8_ = 48.17; *p* < 0.001). There was no significant difference in growth between *Daphnia* inoculated with microbiota from different populations that received the same OTC concentration. *Daphnia* inoculated with microbiota exposed to 100 μg OTC L^−1^ for 7 days grew significantly larger than those inoculated with microbiota exposed to 10 μg OTC L^−1^ for 7 days or no OTC (Fig. 4). *Daphnia* inoculated with microbiota exposed to 100 μg OTC L^−1^ for 23 days and 1 mg OTC L^−1^ for 23 days grew significantly larger than those inoculated with microbiota exposed to 10 μg OTC L^−1^ for 23 days and no OTC. Furthermore, *Daphnia* receiving a microbiota inoculum from 23-day-old populations that were not exposed to OTC were significantly smaller than all other treatments.

**Fig. 5:**
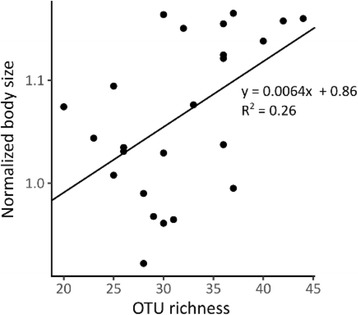
Scatterplot of normalized body size in function of OTU richness. A significant positive correlation between normalized body size and OTU richness was found by fitting a linear regression model (*F*_1,22_ = 7.8, *p* = 0.01)

Tests for differential abundance of OTUs between small and large *Daphnia* indicated that several OTUs were specific for each group (Table 1). Notably, Neisseriaceae sp. had a high mean relative abundance in small *Daphnia* (51.1% ± 36.6%), while either absent or occurring at a very low abundance in large *Daphnia* (0.1% ± 0.1%). Chitinophagaceae sp. and *Rheinheimera* sp. also occurred at a significantly higher abundance in small *Daphnia*. In large *Daphnia*, on the other hand, *Polynucleobacter* sp., *Hansschlegelia* sp., and Xanthobacteraceae sp. occurred at significantly higher abundances. Tests for correlations between α-diversity and host growth showed a significant correlation between the normalized body size of *Daphnia* and the OTU richness of the associated microbiota community (*F*_1,22_ = 7.8, *p* = 0.01) (Fig. 5), but not between normalized body size and Shannon diversity.

**Table 1:** OTUs which were found to have a significantly differential abundance between small *Daphnia* (normalized body size < 1.0) and large *Daphnia* (normalized body size > 1.1). For each OTU the highest assigned taxonomy, mean relative abundance (± standard deviation on relative abundance) and the adjusted *p* value determined calculated with DESeq2 for differential abundance between the two groups is given. Only OTUs which occurred in at least two samples within a group are included

OTU (highest assigned taxonomy)	Mean relative abundance		p-adj.
	Small *Daphnia*	Large *Daphnia*	
Neisseriaceae sp.	51.1% (± 36.6%)	0.1% (± 0.1%)	1.74 × 10^−12^
Chitinophagaceae sp.	2.1% (± 4.7%)	0.2% (± 0.5%)	4.61 × 10^−03^
*Polynucleobacter* sp.	–	1.3% (± 2.6%)	4.61 × 10^−03^
*Hansschlegelia* sp.	–	0.8% (± 1.7%)	1.62 × 10^−03^
Xanthobacteraceae sp.	–	0.3% (± 0.4%)	1.85 × 10^−03^
*Rheinheimera* sp.	0.07% (± 0.09%)	–	7.03 × 10^−03^

## Discussion

The effect of exposing microbiota of *Daphnia magna* to different concentrations of oxytetracycline and the subsequent consequences for community assembly when germ-free *Daphnia* were colonized with the disturbed microbiota were tested. The impact of differences in microbiota assembly on host growth was assessed. Exposure to oxytetracycline was found to cause a concentration-dependent disturbance on the composition of the microbiota which, in turn, affected microbiota assembly and host-microbiota interactions after colonization of germ-free *Daphnia*.

Exposure of the microbiota to oxytetracycline induced shifts in community composition which were dependent on both the concentration of oxytetracycline and the length of exposure. It is well known that the effect of antibiotics on bacterial responses is concentration-dependent, where they can act as either toxins, stress inducers, or cues on receiver bacteria. As responses of specific taxa to different antibiotic concentrations can vary substantially, microbiota communities are likely to respond in a non-linear way to antibiotic gradients [31]. Furthermore, antibiotic exposure can impact the microbiota indirectly through its effect on host physiology (e.g., via host immunity, [3, 32, 33]), which can also be dose-dependent [34]. In this study, an increase in resistance of *Hydrogenophaga* sp. was observed after 23 days of exposure to 100 μg OTC L^−1^, which allowed this OTU to outcompete *Acinetobacter* sp. and dominate the microbiota community. This response was, however, not observed in populations exposed to 1 mg OTC L^−1^. Probably, 100 μg OTC L^−1^ was below the minimal inhibitory concentration for *Hydrogenophaga* sp., allowing for the selection of resistant genotypes over time. *Acinetobacter* sp. was always the most abundant taxon in the microbiota community exposed to the highest oxytetracycline concentration. Although the total number of bacteria was strongly reduced after 7 days of exposure to 1 mg OTC L^−1^, *Acinetobacter* sp. showed a significant increase in antibiotic resistance after 23 days. Members of this genus are well known to be resistant to most of the available antimicrobial agents, and *tet* genes, conferring resistance to oxytetracycline, have been detected in multiple strains. Eckert et al. [35] showed that the *Daphnia* microbiome can readily acquire tet(*A*) genes from the environment and postulated that its biofilm-like structure may facilitate horizontal gene transfer between members of the microbiota. Further follow-up experiments via in vitro growth assays could evaluate antibiotic resistance of these specific taxa and would provide additional support for this conclusion in a more quantitative manner. Interestingly, almost all replicate populations showed highly similar responses to oxytetracycline exposure. Similar patterns were observed in mice, where antibiotic administration caused reproducible changes in the gut microbiota community structure, indicating that these communities can exhibit stereotypical responses if ecological stressors are consistently applied [36]. However, one population exposed to 10 μg OTC L^−1^ showed a discordant response in comparison to other populations exposed to the same level of disturbance, showing that responses of similar communities to stressors can also vary.

In undisturbed populations of this experiment, the microbiota community composition was found to be relatively simple, with only two or three different OTUs belonging to the Proteobacteria dominating the community and a total richness not exceeding 40 OTUs. This relatively simple community structure is in accordance with earlier studies [26, 27, 37]. However, the genus *Limnohabitans*, which was found to be the most abundant taxon in all these studies, was notably absent in this study. This finding indicates that the composition of the *Daphnia* microbiota can vary substantially, at least at lower taxonomic levels. It was observed that the microbiota community composition in undisturbed populations did not remain stable over time but varied in a similar manner in all populations with an increase in Neisseriaceae sp. and a decrease in *Acinetobacter* sp. Possibly, this shift in the community composition is caused by the increased frequency in medium changes compared to stock cultures from which the *Daphnia* originated, causing OTUs which are better adapted to these circumstances to increase in abundance over time. This same response seemed to be intensified after inoculation of undisturbed microbiota in germ-free *Daphnia*, indicating that Neisseriaceae sp. can outcompete other taxa when the total numbers of bacteria are reduced during colonization.

Although local environmental conditions are generally found to be an especially important structuring factor for microbial communities [38], our experiments showed that the membership and relative abundance of specific taxa in the microbiota inoculum affected ecological interactions within the microbiota. Microbiota assembly was found to be very different between germ-free *Daphnia* that were colonized with inocula from microbiota communities experiencing different degrees of oxytetracycline-induced disturbance in *Daphnia*. Although microbiota was inoculated in highly similar environments, differences in community composition could be expected due to changes in membership of specific taxa. Besides not being able to colonize a new host because of its absence in the inoculum, the removal of a species can further affect colonization by other species who are present in the inoculum, as microbiota communities often consist of a complex network of co-dependence [39]. The removal of dominant competitors can, on the other hand, also have strong effects on community structure by allowing for the establishment of a more diverse and even community. In our experiment, Neisseriaceae sp. was found to be a dominant competitor on *Daphnia* inoculated with microbiota showing a low level of disturbance. However, the occurrence of Neisseriaceae sp. was either strongly reduced or completely removed in inocula exposed to higher concentrations of oxytetracycline. This reduction resulted in either the establishment of a more diverse community on *Daphnia* or allowed other taxa to dominate the microbiota in the absence of Neisseriaceae sp. These findings indicate that the composition of the pool of colonizers during horizontal transmission and subsequent ecological interactions between the members of the microbiota can be an important primary structuring factor of the *Daphnia* microbiota, opposed to a strong selection by the host habitat.

Substantial differences were furthermore observed between the microbiota community composition of inoculated *Daphnia* and the inoculum that was administered. The absence of oxytetracycline after inoculation could have enabled susceptible OTUs, for which the growth was suppressed when exposed to oxytetracycline, to increase in abundance after inoculation. This response would be similar to a recovery of the microbiota community after an antibiotic-induced disturbance and could explain the observed differences between the inoculum and the established microbiota [14]. As recovery dynamics of antibiotic-disturbed microbiota have also been found to be dependent on the severity of antibiotic pressure, these could furthermore also account for differences between *Daphnia* inoculated with microbiota exposed to different oxytetracycline concentrations [36]. However, we also observed large differences in microbiota community composition between inocula not exposed to oxytetracycline and the *Daphnia* colonized by these inocula. This indicates that the microbiota assembly mechanisms are strongly affected when germ-free *Daphnia* are inoculated with microbiota. Here, a small amount of microbiota was added to an otherwise sterile environment while under normal conditions, *Daphnia* are colonized from an environment containing a differentiated and abundant bacterial community [37]. The inoculation procedure applied in this experiment is expected to cause a decrease in abundance of potential colonizers alongside a shift in community composition, increasing the relative abundance of *Daphnia*-associated taxa in the environment. This method is expected to especially favor taxa that are strong competitors on *Daphnia* but have otherwise limited dispersal abilities.

Microbiota assembly on *Daphnia* was found to have a significant effect on host growth. Furthermore, differences in microbiota assembly and host growth were related to the concentration of oxytetracycline to which the microbiota inoculum was exposed. Overall, *Daphnia* inoculated with microbiota exposed to higher concentrations of oxytetracycline performed better. In this experiment, oxytetracycline exposure had a strong effect on the abundance of Neisseriaceae sp., both before and after inoculation. Furthermore, this OTU was found to be present at a significantly higher relative abundance on the smallest *Daphnia* than on the largest *Daphnia*. A strong reduction in Neisseriaceae sp. allowed for different taxa to colonize the host, with an increased diversity in some communities. It was found that about a quarter of the variation in *Daphnia* growth could be predicted by microbiota diversity. As respiration rates of bacterial communities are known to be influenced by species richness and composition [39], more diverse communities possibly contain a wider array of metabolic capabilities, allowing for example a better food digestion. The increased abundance of specific taxa could furthermore benefit the host if these are better suited for providing a specific service. Interestingly, in some cases, very disparate communities exerted a similar effect on host growth, indicating for a certain degree of functional redundancy in host-microbiota interactions.

## Conclusion

These experiments show that the *Daphnia* holobiont composition is affected by oxytetracycline exposure, dependent on both the oxytetracycline concentration and the length of exposure. Prolonged exposure resulted in an increased growth of resistant bacteria on *Daphnia*. It has been shown that the composition of the pool of colonizing bacteria during horizontal transmission can substantially influence microbiota assembly of the *Daphnia* holobiont. This factor could also play an important role in structuring the *Daphnia* microbiota natural populations, as different environmental conditions could potentially affect the pool of colonizing bacteria. This, however, remains to be investigated. Holobiont performance was also significantly affected after inoculation, with *Daphnia* receiving inocula with the highest degree of disturbance showing an overall better growth. Furthermore, these results indicate the possibility of manipulating the *Daphnia* microbiota through the composition of the inoculum. Further experiments using cultivated *Daphnia*-associated bacteria, allowing for a higher degree of experimental control, could give a better insight in the complex ecological interactions governing microbiota-assembly and how this affects the holobiont’s fitness.

## Methods

### Cultivation of *Daphnia* and axenic *Chlorella*

Throughout this study, *Daphnia magna* strain NIES (National Institute for Environmental Studies, Tsukuba, Japan) was used for all experiments. Stock cultures of *Daphnia* were kept in 5 L ADaM at 23 °C ± 1 °C under a regime of 16:8 h light:dark. *Daphnia* were fed 1 × 10^5^ cells mL^−1^ of *Chlorella* for the first week and 2 × 10^5^ cells mL^−1^ afterwards. *Daphnia* (both juveniles and eggs) used in subsequent experiments were isolated from stock cultures that were known to have released at least one brood.

Axenic *Chlorella vulgaris* Beijerinck (National Institute of Environmental Studies, Tsukuba, Japan) was grown by inoculating a small amount of cells into a sterile Erlenmeyer containing 200 mL autoclaved MAM medium (0.0025% CaCl_2_·H_2_O; 0.0075% MgSO_4_; 0.0025% NaCl; 0.01% KNO_3_; 0.025% NH_4_NO_3_; 0.2% casamino acids; 0.05% yeast extract; 0.05% malt extract). Cultures were grown for 5 days on a shaking plate at 23 °C under a regime of 16:8 h light:dark. The concentration of *Chlorella* was determined using a CDA-1000 cell counter (Sysmex). Afterwards, cells were collected, washed twice, and resuspended in filtered M4 medium [40]. Harvested cells were stored at 4 °C for a maximum of 2 weeks. Bacterial contamination of algal cultures was tested using a qPCR assay for the detection of 16S rRNA (see further for details).

### Exposure of *Daphnia* populations to OTC

At the start of the experiment, 360 neonate *Daphnia* were collected from laboratory stock cultures. These neonates were randomly divided among aquariums containing 2 L ADaM, resulting in 30 *Daphnia* per aquarium (referred to as a population from hereafter). Each population was assigned to one of four possible treatments: either continuous exposure to one of three different concentrations of oxytetracycline (OTC; oxytetracycline hydrochloride > = 95% [HPLC] crystalline, Sigma) (1 mg L^−1^, 100 μg L^−1^, or 10 μg L^−1^) or a control treatment to which no OTC was added. Preliminary experiments to determine a concentration of OTC with a noticeable impact on bacterial load but without completely removing all bacteria showed that at 1 mg L^−1^ OTC a low number of bacteria was still detectable on *Daphnia* after 48 h of exposure, while no more bacteria were detectable at higher concentrations. Therefore, 1 mg L^−1^ OTC was used as the maximum concentration. Each treatment was set up in triplicate. The experimental populations were placed in a temperature-controlled room at 21 °C under a regime of 16:8 light:dark and were fed daily with *Chlorella* (1.05∙10^5^ cells·mL^−1^ for the first week and 2.1 × 10^5^ cells mL^−1^ afterwards). Every other day, the medium of all populations was refreshed and neonate *Daphnia* were removed. After refreshing the medium, concentrations of OTC were restored by adding the respective amount of OTC to each experimental population. This way, OTC levels were expected to remain relatively constant throughout the experiment, despite the known degradation of OTC [41]. All experimental populations were maintained for 23 days. Samples for the determination of bacterial load and microbiota community composition were taken after 7 and 23 days of exposure alongside determination of the effect of the microbiota on host growth. For this, a total of nine adult *Daphnia* were removed from the experimental populations on each sampling point (see further sections for details on sampling methods for each parameter). To keep the number of *Daphnia* constant throughout the experiment, these removed individuals were subsequently replaced with neonates produced by *Daphnia* within their respective experimental population.

### Inoculation of microbiota communities and determination of host growth

*Daphnia* growth was used as a measure for the effect of inoculated microbiota on host growth, as this trait is known to be strongly affected by the microbiota [25, 27]. Host growth was determined in the absence of antibiotics by re-inoculating germ-free *Daphnia* with microbiota from exposed populations and subsequently measuring their growth. Germ-free *Daphnia* were obtained by disinfecting parthenogenetic eggs from stock cultures by exposing them for 30′ to a 0.25% solution of glutaraldehyde (G7776, Sigma) [26]. These eggs were subsequently rinsed with sterile ADaM, transferred to a six-well plate containing 5 mL of sterile ADaM per well, and incubated at 21 °C and a 16:8 light: dark cycle for 48 h. Afterwards, experimental units were set up by individually transferring a hatched germ-free *Daphnia* to a falcon tube containing 40 mL of sterile ADaM.

Microbiota inoculates were prepared by homogenizing three *Daphnia* from an exposed population in 900 μL of sterile ADaM. To each experimental unit, 100 μl of the appropriate microbial inoculum was added. With a microbial inoculum originating from a single population, five *Daphnia* were inoculated. This resulted in a total of 15 replicates per OTC concentration (5 inoculated *Daphnia* × 3 populations). Every day, each experimental unit was given 1.210^5^ × cells mL^−1^ of axenic *Chlorella*, and survival of each individual was recorded.

After 6 days, a photograph of each *Daphnia* was taken under a stereo microscope and body size was determined using the ImageJ software [42]. To determine the community composition of the microbiota in the inoculation experiment, three *Daphnia* receiving an inoculum from a single experimental population were collected, frozen in liquid nitrogen, and stored at − 80 °C until further processing (see further).

### Determination of bacterial load

A qPCR assay was used to measure the quantitative impact of OTC on the *Daphnia* microbiota after 7 and 23 days of exposure. For this, DNA was extracted from three pooled adult *Daphnia* originating from the same experimental population following Huang et al. [43]. qPCR reactions were performed on a Light Cycler 480 using SYBR Green I Master (Roche) on 10 ng of template. Both the bacterial 16S rRNA gene (forward primer 5′-AGACACGGTCCAGACTCCTAC-3′ and reverse primer 5′-CTTGCACCCTCCGTATTACCG-3′) and *Daphnia magna* RPL32 (forward primer 5′-GACCAAAGGTATTGACAACAGA-3′ and reverse primer 5′-CCAACTTTTGGCATAAGGTACTG-3′) were amplified for 45 cycles (95 °C—10s; 60 °C—20s; 72 °C—5 s). For each gene, four samples with a known copy number were added to create a standard curve. Three technical replicates of each reaction were performed. Absolute quantification of gene copy numbers was calculated from standard curves using the 2nd derivative max method (LightCycler 480 software 1.5.0). For each sample, an index for bacterial load was calculated by dividing the 16S rRNA gene copy number with the RPL32 gene copy number. This normalization procedure was done to compensate for both differences in DNA template and variation in body size, giving the amount of bacteria relative to the amount of *Daphnia* material.

### Sequencing library preparation

The composition of the *Daphnia-*associated bacterial community was characterized for all populations after 7 and 23 days of exposure and for each group of *Daphnia* receiving a microbial inoculum from a single population after testing the effect of an inoculum on host growth (see above). DNA was extracted from three pooled adult *Daphnia* following Huang et al. [43]. Because of initially low bacterial DNA concentrations in some samples, a nested PCR was applied to increase specificity and amplicon yield [16, 44]. First, the full-length 16S rRNA gene was amplified with primers 27F and 1492R on 10 ng of template (94 °C—30s; 50 °C—45 s; 68 °C—90s; 30 cycles) using a high-fidelity *Pfx* polymerase (Life technologies). PCR products were purified using the QIAquick PCR purification kit (Qiagen). To obtain dual-index amplicons of the V4 region, a second amplification was performed on 5 μL of PCR product using primers 515F [45] and a slightly modified version of primer 806R to increase detection of SAR11 bacterioplankton for 30 cycles (94 °C—30s; 55 °C—30s; 68 °C—60s). Both primers contained an Illumina adapter and an 8-nt barcode at the 5′-end. For each sample, PCRs were performed in triplicate, pooled, and gel purified using the QIAquick gel extraction kit (Qiagen). An equimolar library was prepared by normalizing amplicon concentrations with a SequalPrep Normalization Plate (Applied Biosystems) and subsequent pooling. Amplicons were sequenced using a v2 PE500 kit with custom primers [42] on the Illumina Miseq platform, producing 2 × 250-nt paired-end reads.

### Processing of sequencing data

Sequence reads were processed using R 3.3.2 (R Core team, 2016) following Callahan et al. [46]. Sequences were trimmed (the first 10 nucleotides and from position 180 onwards) and filtered (maximum of two expected errors per read) on paired ends jointly. Sequence variants were inferred using the high-resolution DADA2 method which relies on a parameterized model of substitution errors to distinguish sequencing errors from real biological variation and considers each sequence variant as a separate OTU [46]. Chimeras were subsequently removed from the dataset. After filtering, the average number of reads per sample was 96,833 (min. = 19,034 reads, max. = 201,643 reads). Taxonomy was assigned with a naive Bayesian classifier using the RDP v14 training set. OTUs (operational taxonomic unit) with no taxonomic assignment at phylum level or which were assigned as “Chloroplast” were subsequently removed from the dataset. Sequences of dominant OTUs with a low level of taxonomic assignment were further classified using the SINA aligner [47]. A neighbor joining phylogenetic tree was constructed which was used as a starting point for fitting a GTR+G+I maximum likelihood tree. Except for α-diversity calculations, closely related taxa were agglomerated for subsequent analysis at a tree height of 0.1.

### Analysis of microbiota communities

Data on bacterial load showed a non-normal distribution; therefore, differences in bacterial load after the same length of exposure between OTC concentrations and between exposure times within a single OTC concentration were tested using a non-parametric Kruskal-Wallis rank sum test.

As measures for alpha-diversity within different microbiota communities, OTU richness (total number of OTUs present) and Shannon index (taking into account both OTU richness and the relative abundance of OTUs) were calculated for both lengths of exposure in experimental populations and inoculated *Daphnia* using the vegan package in R [48]. Differences in OTU richness and Shannon index between OTC concentrations were tested with a one-way ANOVA and a Tukey HSD test for post hoc comparison. To investigate the beta-diversity between different microbiota communities, weighted UniFrac distances were calculated ([49] this distance metric takes into account the phylogenetic distance between OTUs and their relative abundance within a sample) and plotted using principal coordinates analysis with the phyloseq package in R [50].

### Analysis of host growth

For the body size data, normal distribution and equal variance for each group of *Daphnia* receiving a microbial inoculum from the same population were tested using a Shapiro-Wilk and Bartlett test, respectively. For each length of exposure, differences in *Daphnia* growth between microbiota inocula were analyzed using a nested one-way ANOVA with OTC concentration as a fixed effect and population as a random effect. A Tukey HSD test was used to make post hoc pairwise comparisons.

The differential abundance of specific OTUs between *Daphnia* that show differences in growth was calculated. Growth measurements for *Daphnia* inoculated with microbiota exposed for 7 and 23 days were obtained in separate experiments. In order to correct for this, the body size data was first normalized by dividing each measurement with the median body size of *Daphnia* inoculated with microbiota from the control treatment of the same exposure time. After normalization, inocula were selected which gave “large” *Daphnia* (mean normalized body size > 1.1; *n* = 9) and inocula which gave “small” *Daphnia* (mean normalized body size < 1; *n* = 6). Differential abundance of OTUs between large and small *Daphnia* was tested using DESeq2 [51, 52].

To test for the correlation between α-diversity (OTU richness and Shannon index) in a microbiota community and the mean normalized body size of *Daphnia* from which this community was obtained, a linear regression model was fitted with normalized body size as dependent variable and either OTU richness or Shannon index as explanatory variable.
